# Involvement of *SIX9* in Growth and Pathogenicity in *Fusarium oxysporum* f. sp. *fragariae*

**DOI:** 10.3390/jof12070475

**Published:** 2026-06-29

**Authors:** Long Li, Wenbo Yang, Chengxing Mao, Yahui Liu, Chuanqing Zhang

**Affiliations:** 1 College of Advanced Agricultural Sciences, Zhejiang Agriculture and Forest University, Hangzhou 311600, China; 18368856884@163.com (L.L.); wbyang0811@163.com (W.Y.); zafumaocx@163.com (C.M.); 2 Extension Centre of Agriculture Technology of Jiande, Hangzhou 311400, China

**Keywords:** strawberry, *Fusarium oxysporum*, *SIX* gene, mutant, pathogenicity, ROS

## Abstract

Strawberries, as an important economic crop, are widely planted worldwide. *Fusarium oxysporum*, belonging to FOSC (*Fusarium oxysporum* species complex), is widely present in plants. Among them, *Fusarium oxysporum* f. sp. *fragariae* (*Fof*) is one of the most important pathogens on strawberry and has pathogenic specificity toward strawberry hosts. In recent years, diseases caused by *Fof* have seriously threatened the strawberry industry. Secreted in Xylem (*SIX*) genes play important and different roles in *F. oxysporum*. In this study, we knocked out *SIX9* in Fof to analyze its functions. The mycelial growth rate of *ΔFofSIX9* was significantly lower than that of the wild type, but the difference in spore production was not significant. The pathogenicity of *ΔFofSIX9* toward four different representative strawberry varieties was significantly reduced, manifested by the decrease in the severity of plant wilt, root rot, and crown rot. In addition, compared to the wild type, the activities of superoxide dismutase (SOD) and catalase (CAT) in *ΔFofSIX9*-infected plants were significantly increased, while the content of malondialdehyde (MDA), hydrogen peroxide (H_2_O_2_), and superoxide anion (O_2_^−^) were significantly decreased. So *∆FofSIX9* could reduce the pathogenicity of the wild type by affecting the host plant’s defense response against infection of *Fof*.

## 1. Introduction

Strawberry is a perennial herbaceous evergreen fruit crop widely cultivated in the world [1]. According to the latest database of the Food and Agriculture Organization of the United Nations (FAO), the strawberry planting area in China in 2022 was 105,000 ha, with a yield of 3.35 million tons, accounting for about 80% of the global total production and ranking first in the world for several consecutive years. Strawberry contains rich natural antioxidants such as high vitamin C, and has a high nutritional and medicinal value, significantly increasing antioxidant capacity in human serum [2] and reducing pain and inflammation in obese adults suffering knee osteoarthritis [3].

The fungi belonging to the *Fusarium oxysporum* species complex (FOSC) are considered major plant pathogens worldwide. According to the records, many species (about 106 species) within FOSC are formae speciales, meaning they cause vascular wilts only on specific host plants [4]. Plant-pathogenic *F. oxysporum* exhibits high host specificity, and the names of formae speciales are usually defined based on their specific hosts (i.e., *F. oxysporum* f. sp. *niveum*, *F. oxysporum* f. sp. *ciceris*, *F. oxysporum* f. sp. *vasinfectum*) [5,6,7]. *F. oxysporum* f. sp. *fragariae* (*Fof*), the forma specialis on strawberry [8,9,10,11], is an example of a forma specialis with a complicated phylogeny and a poorly defined effector profile [12] and it causes systemic wilt of strawberry, infecting the roots and entering the xylem [13]. It was reported that *Fof* was one of the most important pathogens threatening strawberry production in China and many other regions [8,9,14]. Therefore, it is urgent to explore the pathogenic mechanism of Fof.

The evidence of lateral gene transfer in *F. oxysporum* provides a stronger basis for studying the pathogenicity-related genes [15]. The host range and specificity of *F. oxysporum* are dictated by genes located on pathogenicity-associated genomic regions [16,17,18,19]. At present, the most in-depth research on the effect of genes of plant pathogens is mainly in fungi including *F. oxysporum*, of which Secreted in Xylem (*SIX*) genes are the most typical [20]. Initially, *SIX* genes were considered to be limited to *F. oxysporum* f. sp. *lycopersici* (Fol), but later, homologs were identified in other formae speciales as well [21]. Li et al. [22] found that *SIX1* was a prerequisite for complete virulence of the pathogen *F. oxysporum* f. sp. *conglutinans* (*Focon*) on cabbage. Rep et al. [23] also found that *SIX1* was a prerequisite for virulence of the Fo*l* on tomato. Thatcher et al. [24] found that in Fo-5176, *SIX4* deletion mutants resulted in reduced disease symptoms. Similarly, Kashiwa et al. [25] found that deletion of *SIX4* led to a reduction in disease severity on both resistant and susceptible cabbage plants compared to the *SIX4*-complemented and wild-type strains in *Focon*. Meanwhile, van Dam et al. [26] found that Fol-*ΔSIX5* displayed an apparent reduction in disease symptoms and Gawehns et al. [27] found that knockout mutants of *SIX6* in Fol and *F. oxysporum* f. sp. *radicis-cucumerinum* (*Forc*) confirmed the role of *SIX6* in pathogenicity. Only *SIX9* and *SIX8* were detected in all 33 tested isolates of *F. oxysporum* f. sp. *palmarum*, while *SIX10* was present in seven of these isolates [28].

Prior to this study, our research group systematically examined the *SIX10*-Fof interaction dynamics [29]. Despite growing interest in fungal pathogenesis mechanisms, well-documented studies on the functional relationship of *SIX9* with *Fof* remain conspicuously absent. The elucidation of roles of *SIX* in *F. oxysporum* has significant meaning in establishment of a molecular framework and management of relative diseases. This study successfully generated a *SIX9* mutant and conducted comprehensive phenotypic characterization alongside pathogenicity assays, with parallel evaluations in wild-type strains, to establish a theoretical framework for understanding SIX9 and *Fof*.

## 2. Materials and Methods

### 2.1. Media

PDA (1 L): Potato 200 g, glucose 20 g, and agar 20 g.

Oatmeal agar (OA, 1 L): Total of 30 g of oatmeal and 16 g of agar.

Minimal medium (MM, 1 L): Total of 30 g of sucrose, 1 g of KH_2_PO_4_, 0.5 g of MgSO_4_·7H_2_O, 0.01 g of FeSO_4_·7H_2_O, 0.5 g of KCl, 2 g of NaNO_3_, and 200 µL of 1× trace element [29].

Water agar medium (WAM, 1 L): Total of 16 g of agar.

### 2.2. Determination of Growth, Spore Production, and Spore Germination of Pathogen

The wild-type strain H6, *F. oxysporum* f. sp. *fragariae* (Fof), was identified in our previous research [29].

Mycelial plugs (5 mm diameter) were aseptically inoculated onto new PDA plates and incubated in darkness at 28 °C. Following incubation for the designated period, colony diameter was measured. For each treatment, three colonies were adopted and the total tests repeated twice. The mycelial growth rate and spore production rate were determined after 7 days of cultivation.

The spore suspension was prepared through static sporulation methodology [30]. Three cultures of each fungal strain were incubated at 28 °C for 7 days. Each colony was hydrated with 5 mL sterile ddH_2_O (double-distilled water), followed by gentle mycelial abrasion using a sterile glass slide to liberate spores. The resulting suspension was sequentially filtered through triple-layered lens papers, homogenized by vortex mixing, and precisely aliquoted (8 μL) via micropipette. Spore density quantification was performed with over 5 distinct microscopic fields counted per biological replicate.

The spore germination assay was performed according to published methodology [31]. Briefly, conidial suspensions were standardized to 1 × 10^6^ spores/mL using ddH_2_O. Aliquots (20 μL) were aseptically pipetted onto WAM evenly. Plates were incubated under controlled conditions (28 °C, darkness) with three replicates per fungal isolate. Germination was quantified by microscopic examination in 14 h incubation. For statistical robustness, over 100 spores were enumerated per microscopic field. Germination rates were calculated as Germination rate (%) = (number of spores germinated/total number of observed spores) × 100.

### 2.3. Construction of Fof16561 Deletion Mutants

The *Fof16561* deletion mutant (*ΔFof16561*) was constructed by using the double-joint (DJ) PCR approach [32]. First, the upstream (966 bp) and downstream (983 bp) sequences of *Fof16561* were amplified using primer pairs *Fof16561*-UP-F/*Fof16561*-UP-R and *Fof16561*-DOWN-F/*Fof16561*-DOWN-R (Table 1), respectively. Meanwhile, a 1349 bp fragment encoding the hygromycin B phosphotransferase (HPH) cassette, which contains the hygromycin-resistant gene and the trp C promoter, was amplified using primer pair HPH-F/HPH-R. Secondly, to amplify accurately a fragment containing upstream HPH cassette, the three amplicons were added in a PCR reaction tube and fused using the nested primer pair *Fof16561*-Nest-F/*Fof16561*-Nest-R (Table 1).

Finally, the polyethylene glycol (PEG)-mediated Fof protoplast transformation was used to obtain the mutants with deletion, of which the procedures and details are described in our previous study [29].

### 2.4. The Effect of Abiotic Stress on Deletion Mutants

For abiotic stress simulation experiments, Congo red was used for acid stress, KCl was used for salt stress, SDS was used for cell membrane stress, glucose was used for nutritional stress and NaCl was used for osmotic stress [29]. All stress treatments were implemented through assessment of the mycelial growth inhibition rates on PDA plates respectively amended with 0.2 g/L Congo red, 1 M KCl, 0.5 g/L SDS, 1 M glucose, and 1 M NaCl using the standardized inoculation protocol detailed in Section 2.2, with three replicates per condition.

### 2.5. Pathogenicity Testing

Conidia suspension with 1 × 10^6^ spores/mL was prepared by the static spore production method and used as the inoculation [30]. The pathogenicity of the strains *ΔFof16561* and wild-type H6 were conducted using the root soaking method as described by Fang et al. [31]. Briefly, the roots of strawberry seedlings were soaked in conidia suspension or sterile water for 20 min, then the incubated seedlings were planted and placed in a chamber at 28 °C with a 12 h photoperiod and 85% relative humidity for 15 days. The result of pathogenicity was evaluated based on the degree of plant wilt and root rot caused by inoculation as previously described [33,34].

To test the pathogenicity of the strains *ΔFof16561* and wild-type H6 at the crown of the strawberry, the spray inoculation method was performed [29]. Superficial wounds in the epidermis at the crown were created by using sterilized insect needles, followed by spraying with 5 mL conidia suspension (5 × 10^6^ spores). Then the incubated seedlings were placed in a chamber at 28 °C with a 12 h photoperiod and 85% relative humidity. After 15 days, the crown of each seedling was cut open for observing the damage, and the rating scale for the crown was classified as previously described [35]. Each treatment included three seedlings in each inoculation method and the tests were repeated twice.

By observing the disease situation of the interaction between the pathogen and strawberry, referring to the grading standards of the three parts mentioned above, the disease level was recorded, and the disease index was calculated according to the following formula:

Disease index = ∑ (number of plants × number of degree in symptoms)/(total number of plants) × (maximum degree in symptoms) × 100 [34].

### 2.6. Determination of Plant Physiological and Biochemical Indicators

The 40-day old strawberry healthy seedlings were inoculated using the root inoculation method as described in Section 2.5 with 1 × 10^6^ spores/mL suspensions of *ΔFof16561* and wild-type H6. Samples were taken randomly at the early stage (1 d), middle stage (7 d), and late stage (14 d) of infection. Three seedling replicates were adopted for each treatment at each time point. After the sampling was completed, the seedlings were stored in 80 °C and analyzed by Wuhan ProNets Testing Technology Co., Ltd. (Wuhan, China), for SOD, POD, CAT, MDA, H_2_O_2_, O_2_^−^, soluble sugar, soluble protein, and proline.

### 2.7. Statistical Analysis of Data

We used SPSS 29.0 and Prism 10 to analyze and process the statistics. Differences in measurement means were analyzed using ANOVA and Fisher’s LSD tests.

## 3. Results

### 3.1. SIX Homologous Gene Evolutionary Tree Analysis

**Figure 1:**
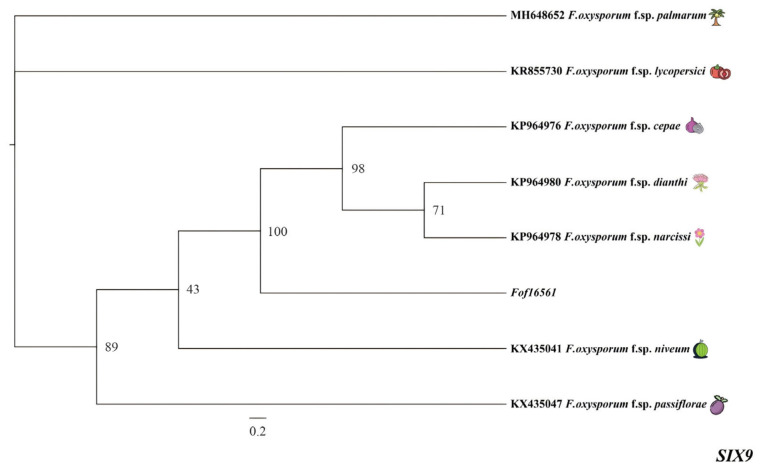
Cladogram of similarity between the   *Fof*  strain and the  *SIX9*  genes of other host-specialized strains.  The scale bar shows 0.2 expected changes per site.

Plant-pathogenic *F. oxysporum* has destructive effects on its host plants and shows a high degree of host specificity. The different formae speciales of *F. oxysporum* harbor different sets of *SIX* genes. Thus far, no studies other than our previous work on SIX10 [29] have been published on the *SIX* genes of Fof. Based on the sequence homology analysis of the *SIX9* gene of *F. oxysporum* from various hosts, *Fof16561* from strawberry forma specialis was clustered with that from palm, tomato, onion, carnation and narcissus formae speciales, suggesting that the *Fof16561* and the other five formae speciales of *F. oxysporum* are closely related; therefore, we conclude that *Fof16561* is the *SIX9* gene (Figure 1).

### 3.2. Deletion of Fof16561 in Fof

**Figure 2:**
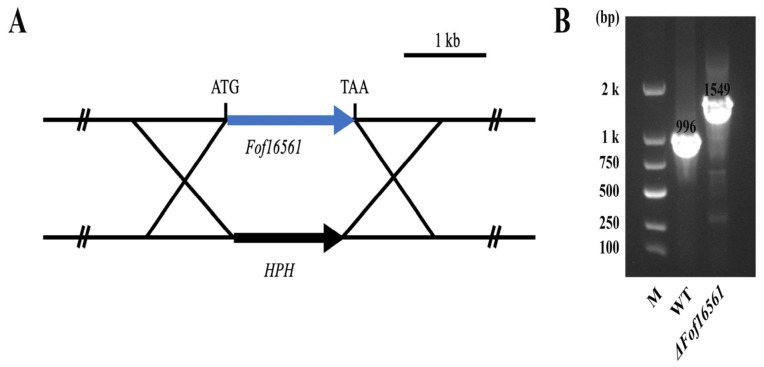
Generation and identification of *Fof16561* deletion mutant by gene replacement. (**A**) Schematic representation of the *Fof16561* replacement strategy. (**B**) PCR verification of the *Fof16561* deletion mutation. Note: HPH is hygromycin B phosphotransferase, 1 kb = 1000 bp, and the arrow indicates from upstream to downstream.

One *Fof16561* gene deletion mutant (*ΔFof16561*) was obtained. The primer pair *Fof16561*-ID-F/*Fof16561*-ID-R (Table 1) amplified 1549 bp from *Fof16561* (Figure 2B).

### 3.3. Growth, Spore Production, and Spore Germination of ΔFof16561

**Figure 3:**
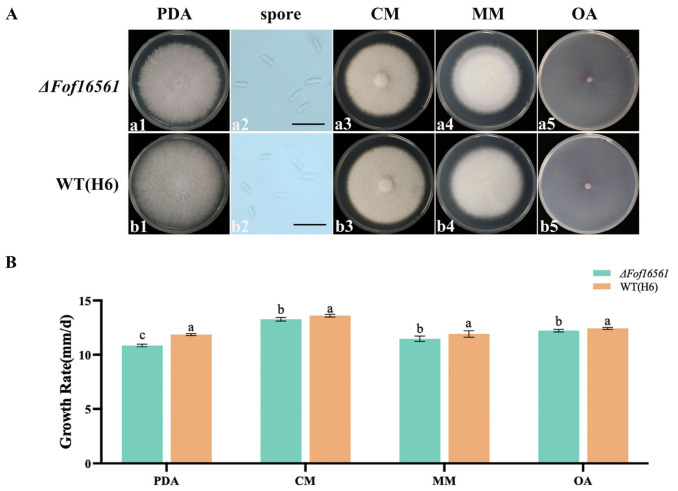
(**A**) Colony morphology of wild-type H6 and *ΔFof16561* on the PDA (**a1**,**b1**) and conidia (**a2**,**b2**) (scale bar: (**a2**,**b2**) = 20 µm), CM (**a3**,**b3**), MM (**a4**,**b4**), and OA (**a5**,**b5**). (**B**) Analysis of growth rate differences among H6 and *ΔFof16561* on different culture mediums. Bars denote the standard errors of tested repeats. Values with the same letters were not statistically different (*p* > 0.05) according to the least significant difference (LSD) test.

The colony morphology of the *ΔFof16561* was similar to that of the wild-type strain H6: the colonies on PDA were flat with the entire margin, with a light purple surface and white margin (Figure 3A). However, knocking out the *SIX9* gene negatively affected the growth of *Fof*. The growth rate of *ΔFof16561* was reduced compared to H6 on all four tested media, reaching 0.91-, 0.93-, 0.94-, and 0.97-fold that of H6 on PDA, CM, MM, and OA, respectively (Figure 3B).

**Figure 4:**
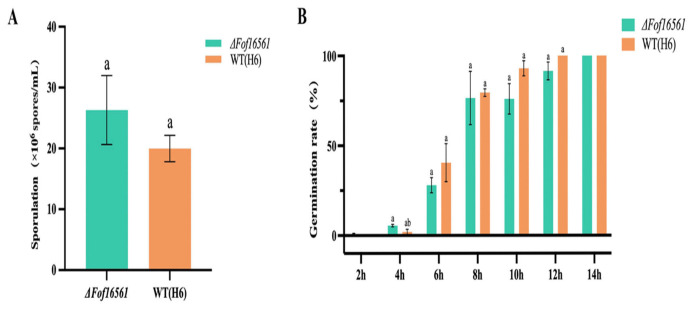
(**A**) The sporulation difference analysis of H6 and *ΔFof16561*. (**B**) The germination rate difference analysis of H6 and *ΔFof16561*. Bars denote the standard errors of tested repeats. Values with the same letters were not statistically different (*p* > 0.05) according to the least significant difference (LSD) test.

There was no significant difference in spore production and germination rate from 6 h to 14 h between the *ΔFof16561* and wild-type strain (Figure 4).

### 3.4. Effect of Abiotic Stress on ΔFof16561

**Figure 5:**
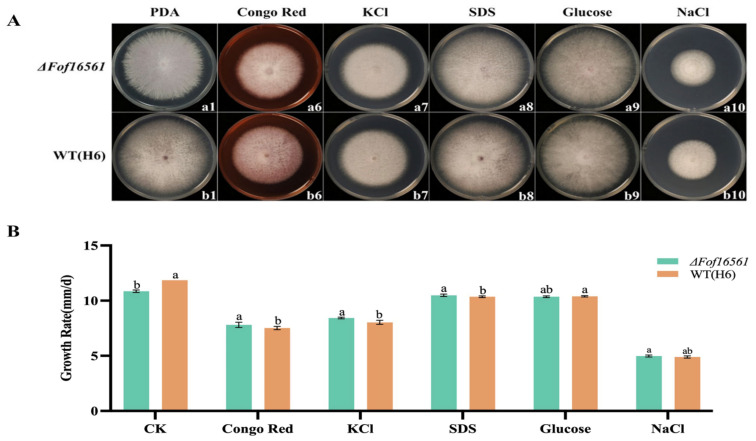
(**A**) Colony morphology of wild-type H6 and *ΔFof16561* on the PDA (**a1**,**b1**), Congo red (**a6**,**b6**), KCl (**a7**,**b7**), SDS (**a8**,**b8**), glucose (**a9**,**b9**) and NaCl (**a10**,**b10**). (**B**) Analysis of growth rate differences among H6 and *ΔFof16561* on different culture mediums. Bars denote the standard errors of tested repeats. Values with the same letters were not statistically different (*p* > 0.05) according to the least significant difference (LSD) test.

Compared to the wild-type strain H6, the sensitivities of *ΔFof16561* to Congo red, KCl and SDS were significantly reduced. However, the sensitivities of the *ΔFof16561* and H6 to glucose and NaCl were not significantly different (Figure 5).

### 3.5. Effect of ΔFof16561 on Pathogenicity

Pathogenicity experiments were conducted through two evaluation methods (root and crown inoculation) on four strawberry varieties, Fenyu, Black Pearl, Lifeng, and Yuexiu. In this section, CK is a blank control, indicating that no disease was observed on the plant, roots, or crown.

**Figure 6:**
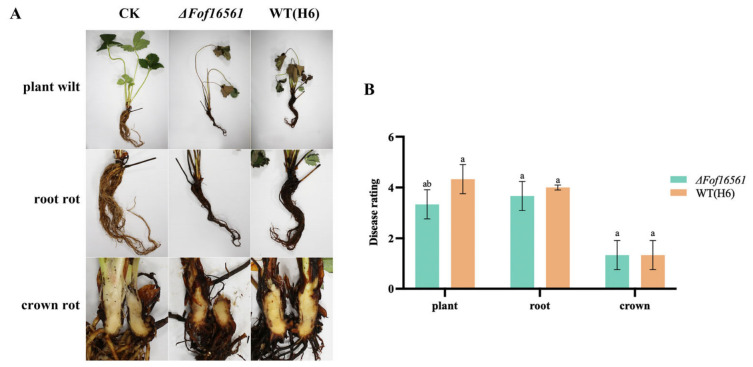
Pathogenicity of *ΔFof16561* and wild-type strain H6 on Fenyu with root inoculation method. (**A**) Plant phenotype diagram. (**B**) Pathogenicity difference analysis of H6 and *ΔFof16561*. Bars denote the standard errors of tested repeats. Values with the same letters were not statistically different (*p* > 0.05) according to the least significant difference (LSD) test.

**Figure 7:**
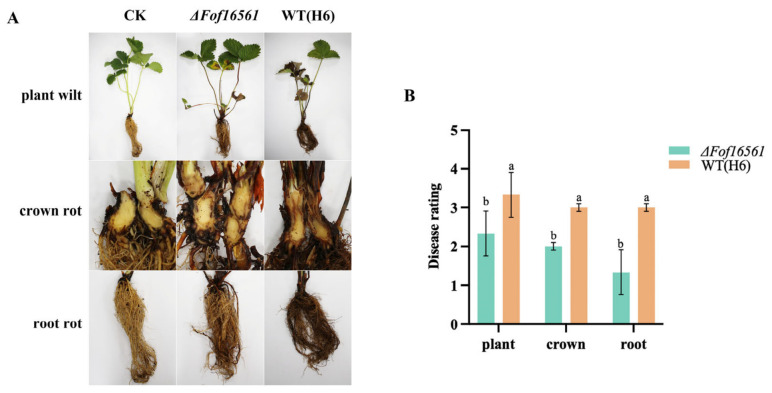
Pathogenicity of *ΔFof16561* and wild-type strain H6 on Fenyu with crown inoculation method. (**A**) Plant phenotype diagram. (**B**) Pathogenicity difference analysis of H6 and *ΔFof16561*. Bars denote the standard errors of tested repeats. Values with the same letters were not statistically different (*p* > 0.05) according to the least significant difference (LSD) test.

Strawberry variety of Fenyu: The root inoculation with *ΔFof16561* reduced pathogenicity on the plant, root, and crown to 0.77, 0.90, and 1.00 times that of H6, respectively, but the differences were not significant (Figure 6). However, crown inoculation with *ΔFof16561* significantly reduced pathogenicity to 0.70, 0.67, and 0.43 times that of H6 for the plant, crown, and root, respectively (Figure 7).

**Figure 8:**
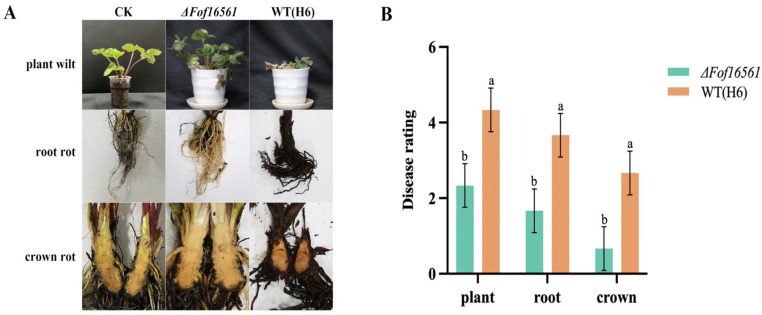
Pathogenicity of *ΔFof16561* and wild-type strain H6 on Black Pearl with root inoculation method. (**A**) Plant phenotype diagram. (**B**) Pathogenicity difference analysis of H6 and *ΔFof16561*. Bars denote the standard errors of tested repeats. Values with the same letters were not statistically different (*p* > 0.05) according to the least significant difference (LSD) test.

**Figure 9:**
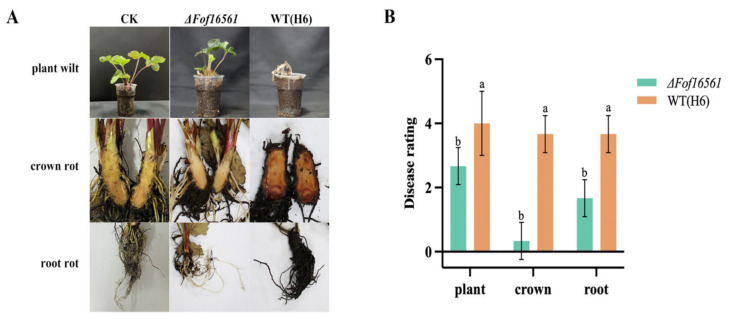
Pathogenicity of *ΔFof16561* and wild-type H6 on Black Pearl with crown inoculation method. (**A**) Plant phenotype diagram. (**B**) Pathogenicity difference analysis of H6 and *ΔFof16561*. Bars denote the standard errors of tested repeats. Values with the same letters were not statistically different (*p* > 0.05) according to the least significant difference (LSD) test.

Strawberry variety of Black Pearl: Root inoculation with *ΔFof16561* reduced disease severity on the plant, root, and crown to 0.53, 0.44, and 0.23 times that of H6, respectively (Figure 8). Crown inoculation with *ΔFof16561* reduced disease severity on the plant, crown, and root to 0.65, 0.08, and 0.44 times that of H6, respectively (Figure 9). All reductions were statistically significant compared to the wild type.

**Figure 10:**
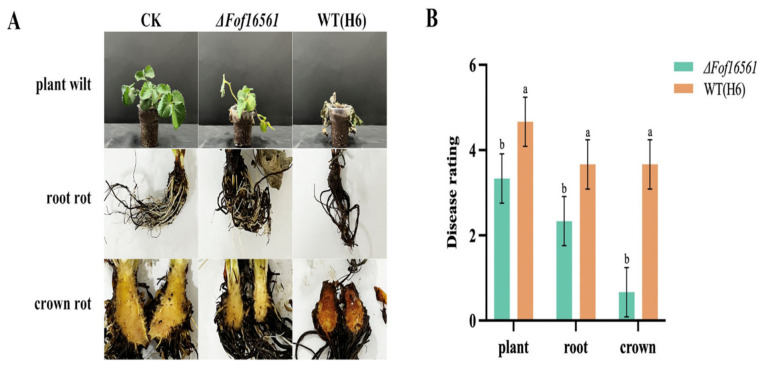
Pathogenicity of *ΔFof16561* and wild-type H6 on Lifeng with root inoculation method. (**A**) Plant phenotype diagram. (**B**) Pathogenicity difference analysis of H6 and *ΔFof16561*. Bars denote the standard errors of tested repeats. Values with the same letters were not statistically different (*p* > 0.05) according to the least significant difference (LSD) test.

**Figure 11:**
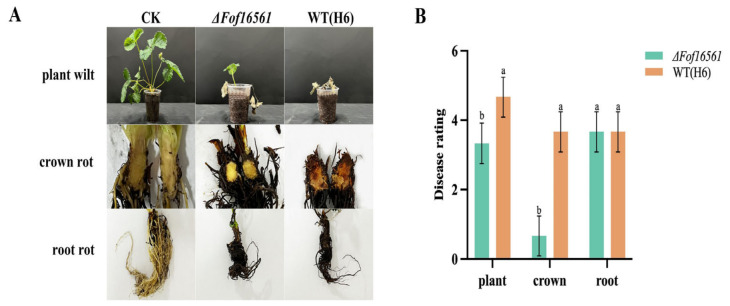
Pathogenicity of *ΔFof16561* and wild-type strains on Lifeng with crown inoculation method. (**A**) Plant phenotype diagram. (**B**) Pathogenicity difference analysis of H6 and *ΔFof16561*. Bars denote the standard errors of tested repeats. Values with the same letters were not statistically different (*p* > 0.05) according to the least significant difference (LSD) test.

Strawberry variety of Lifeng: Root inoculation with *ΔFof16561* reduced disease severity on the plant, root, and crown to 0.71, 0.64, and 0.17 times that of H6, respectively (Figure 10). Crown inoculation with *ΔFof16561* reduced disease severity on the plant, crown, and root to 0.71, 0.17, and 1.00 times that of H6, respectively (Figure 11). All reductions were statistically significant compared to the wild type, except for root severity following crown inoculation, which showed no difference.

**Figure 12:**
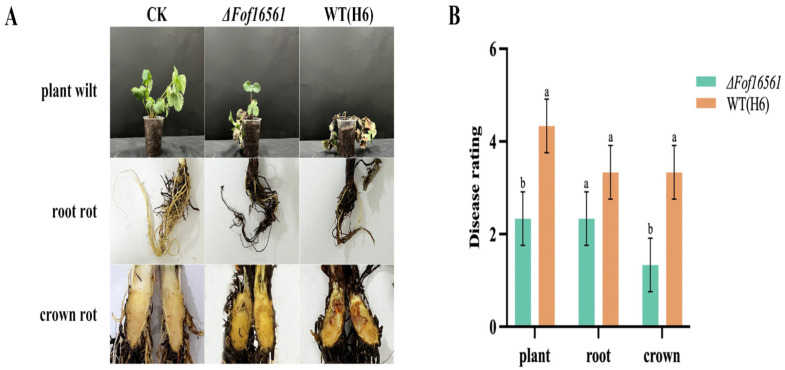
Pathogenicity of *ΔFof16561* and wild-type strains on Yuexiu with root inoculation method. (**A**) Plant phenotype diagram. (**B**) Pathogenicity difference analysis of H6 and *ΔFof16561*. Bars denote the standard errors of tested repeats. Values with the same letters were not statistically different (*p* > 0.05) according to the least significant difference (LSD) test.

**Figure 13:**
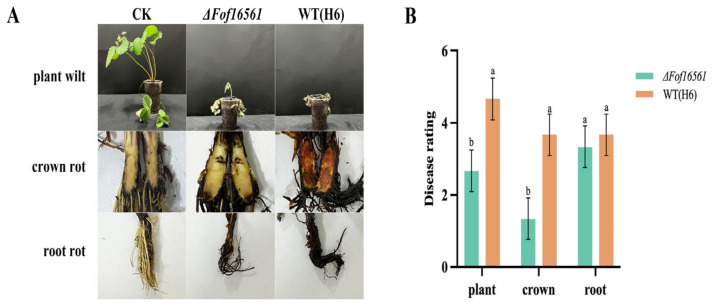
Pathogenicity of *ΔFof16561* and wild-type strains on Yuexiu with crown inoculation method. (**A**) Plant phenotype diagram. (**B**) Pathogenicity difference analysis of H6 and *ΔFof16561*. Bars denote the standard errors of tested repeats. Values with the same letters were not statistically different (*p* > 0.05) according to the least significant difference (LSD) test.

For the strawberry variety of Yuexiu, root inoculation with *ΔFof16561* reduced disease severity on the plant, root, and crown to 0.53, 0.69, and 0.39 times that of H6, respectively (Figure 12). Crown inoculation with *ΔFof16561* reduced disease severity on the plant, crown, and root to 0.56, 0.36, and 0.91 times that of H6, respectively (Figure 13). The reductions in plant wilt and crown rot were statistically significant for both inoculation methods, whereas root rot severity did not differ significantly from the wild type.

### 3.6. Effect of ΔFof16561 on Resistant Responses of Strawberry Plants

We inoculated sterile water, *ΔFof16561*, and wild-type strains separately on strawberry plants, and measured the contents of nine factors of stress in the leaves at the early stage (1 d), middle stage (7 d), and late stage (14 d) of infection.

**Figure 14:**
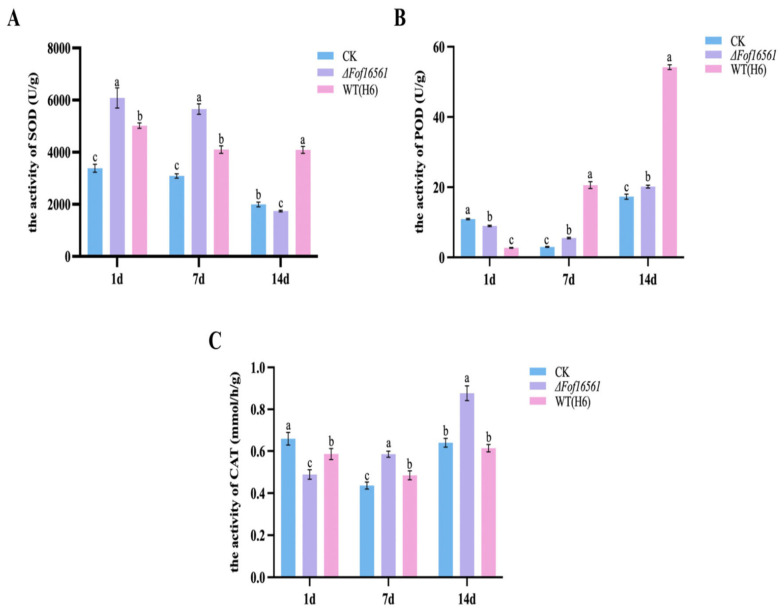
Effects on the SOD, POD and CAT activities of strawberry leaves. (**A**) SOD. (**B**) POD. (**C**) CAT. Data are represented as means ± standard error from *n* = 3 replicates. Values with the same letters were not statistically different (*p* > 0.05) according to the least significant difference (LSD) test. CK is treated with sterile water using the same inoculation method.

Superoxide dismutase (SOD) catalyzes the dismutation of O_2_^−^ into H_2_O_2_ and O_2_, serving as the first line of defense for ROS clearance. It plays an important role in scavenging oxygen free radicals. During the early (1 d) and middle (7 d) stages of infection, SOD had the highest activity in *ΔFof16561*, followed by wild type; CK had the lowest activity and there were significant differences among them. At the end of infection (14 d), the activity of SOD in plants inoculated with *ΔFof16561* was lowest compared to that of un-inoculated CK and the wild type (Figure 14A). We speculated knockout of *SIX9* affected the interactions between *Fof* and the host.

Peroxidase (POD) is widely present in animals, plants, and microorganisms, catalyzing the reaction of H_2_O_2_ with phenolic or amine substrates to produce H_2_O. In the early stage of infection (1 d), POD had the highest activity in CK, followed by *ΔFof16561*, and the wild type had the lowest activity, with significant differences among the three. During the mid stage (7 d) and late stage (14 d) of infection, the POD activity of the wild type was significantly higher than that of *ΔFof16561* and CK. In the late stage of infection, the activity of POD of the wild type reached its highest level. As the infection time progressed, the activity of POD in CK and *ΔFof16561* showed a trend of first decreasing and then increasing, while the activity of POD in the wild type gradually increased (Figure 14B).

Catalase (CAT) is widely present in animals, plants, microorganisms, and cultured cells, and is the most important H_2_O_2_ scavenging enzyme. It decomposes H_2_O_2_ into H_2_O and O_2_, playing an important role in reactive oxygen species scavenging systems. In the early stage of infection (1 d), CAT was CK >> wild type >> *ΔFof16561*. In the middle stage of infection (7 d), it was *ΔFof16561* >> wild type >> CK, and in the late stage of infection (14 d) it was *ΔFof16561* >> CK > wild type. At the late stage of infection (14 d), the CAT content of *ΔFof16561* reached its highest level. As the infection time progressed, the activity of CAT in CK and wild type showed a trend of first decreasing and then increasing, while the activity of CAT in *ΔFof16561* gradually increased (Figure 14C).

**Figure 15:**
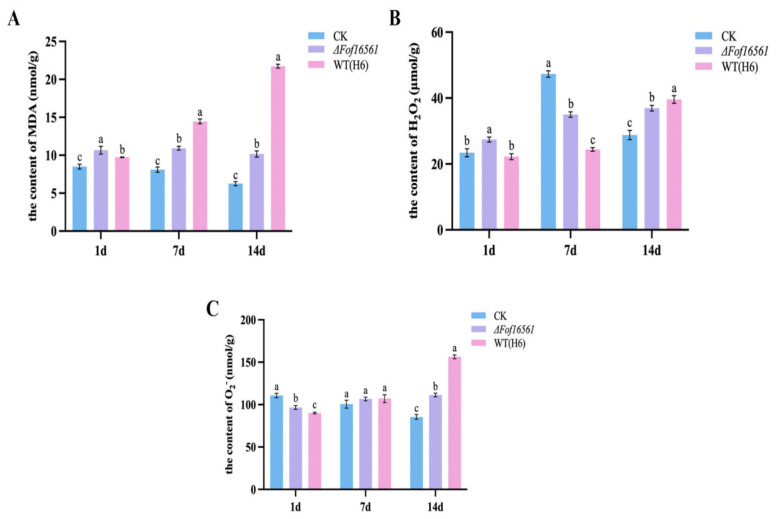
Effects on the MDA, H_2_O_2_ and O_2_^−^ content of strawberry leaves. (**A**) MDA. (**B**) H_2_O_2_. (**C**) O_2_^−^. Data are represented as means ± standard error from *n* = 3 replicates. Values with the same letters were not statistically different (*p* > 0.05) according to the least significant difference (LSD) test. CK is treated with sterile water using the same inoculation method.

Malondialdehyde (MDA) is one of the main end products of lipid peroxidation in plants under stress, widely used as a biomarker of oxidative damage, and its content is positively correlated with the degree of cell membrane damage. In the early stage of infection (1 d), the MDA levels in *ΔFof16561*-inoculated plants remained relatively stable and were significantly higher than those in the wild type and CK. In the middle stage of infection (7 d) and late stage (14 d), the content of MDA in the wild type was significantly higher than that in *ΔFof16561* and CK. In the late stage of infection (14 days), the content of MDA in the wild type reached its highest. As the infection time progressed, the content of MDA in WT continuously and significantly increased (Figure 15A), while the content of MDA for *ΔFof16561* was partially stable and significantly higher than that of CK. These results might suggest sustained lipid peroxidation induced by *ΔFof16561* compared to healthy plants.

Hydrogen peroxide (H_2_O_2_) and superoxide anion (O_2_^−^) are core members of the plant reactive oxygen species (ROS) family, serving as inducers of oxidative damage and key mediators of signal transduction. In the early stage of infection (1 d), the content of H_2_O_2_ in *ΔFof16561* was the highest, significantly higher than that in CK and wild type, and there was no significant difference between the CK and wild type. During the mid stage of infection (7 d), the content of H_2_O_2_ in CK was the highest, significantly higher than that of *ΔFof16561* and the wild type. At the end of infection (14 d), the wild type had the highest content of H_2_O_2_, significantly higher than *ΔFof16561* and CK. During the mid stage of infection (7 d), the content of H_2_O_2_ in CK reached its highest level. As the infection time progressed, the content of H_2_O_2_ in CK first increased and then decreased, while the content of H_2_O_2_ in *ΔFof16561* and wild type gradually increased (Figure 15B).

The content of superoxide anion (O_2_^−^) in CK was significantly higher than that in *ΔFof16561* and wild type at the early stage of infection (1 d). During the mid stage of infection (7 d), the wild type had the highest content of O_2_^−^, followed by *ΔFof16561*, while the CK had the lowest content, and there was no significant difference among the three. At the end of infection (14 d), the content of O_2_^−^ in the wild type was significantly higher than that in *ΔFof16561* and CK. At the end of infection (14 d), the content of O_2_^−^ in the wild type reached its highest. As the infection time progressed, the content of O_2_^−^ in CK gradually decreased, while the content of O_2_^−^ in *ΔFof16561* and wild type gradually increased (Figure 15C).

**Figure 16:**
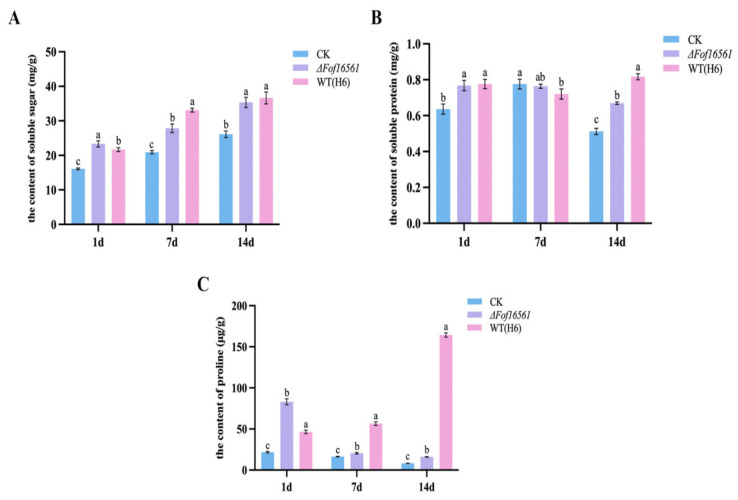
Effects on the soluble sugar, soluble protein and proline content of strawberry leaves. (**A**) soluble sugar. (**B**) Soluble protein. (**C**) Proline. Data are represented as means ± standard error from *n* = 3 replicates. Values with the same letters were not statistically different (*p* > 0.05) according to the least significant difference (LSD) test. CK is treated with sterile water using the same inoculation method.

Soluble sugar is an important osmoregulatory substance and energy source in plants, primarily responsible for maintaining cell osmotic pressure, providing energy, and acting as signaling molecules. In the early stage of infection (1 d), the content of soluble sugar in *ΔFof16561* was significantly higher than in the wild type and CK. In the middle stage of infection (7 d), the content of soluble sugar in the wild type was significantly higher than in *ΔFof16561* and CK. In the late stage of infection (14 d), the content of soluble sugar in the wild type was higher than in *ΔFof16561* but there was no significant difference between them. Notably, the content of soluble sugars in CK was always significantly lower than in the wild type and *ΔFof16561*. In the late stage of infection (14 d), the content of soluble sugars in the wild type reached its highest. As the infection time progressed, the content of soluble sugars in CK, *ΔFof16561* and wild type gradually increased (Figure 16A).

Soluble proteins are involved in the metabolic regulation, stress adaptation, and defense response of plants, stabilizing cell structure, including heat shock proteins (HSPs), disease-related proteins (PRs), and so on. In the early stage of infection (1 d), the content of soluble proteins in the CK was lowest and there was no significant difference between wild type and *ΔFof16561*. During the mid stage of infection (7 d), the soluble protein content in CK was the highest, followed by *ΔFof16561*. The content in the wild type was the lowest, and *ΔFof16561* showed no significant difference in soluble protein content compared to CK or wild type. The difference between CK and wild type was significant. In the late stage of infection (14 d), the content of soluble proteins in plants inoculated with the wild-type H6 was significantly higher than that in the CK and *ΔFof16561*. The content of soluble protein in the wild type reached its highest. As the infection time progressed, the content of soluble proteins in CK increased first and then decreased, while the content of soluble proteins in *ΔFof16561* gradually decreased (Figure 16B).

Proline is one of the most abundant compatible solutes accumulated by plants in stress, with functions such as osmotic regulation, antioxidant activity, and molecular chaperoning. In the early stage of infection (1 d), the content of proline in *ΔFof16561* was significantly higher than that in the wild type and CK. In the middle stage of infection (7 d) and late stage (14 d), the content of proline in the wild type was significantly higher than that in *ΔFof16561* and CK. As the infection time progressed, the content of proline in CK and *ΔFof16561* gradually decreased, but the content of proline in the wild type gradually increased (Figure 16C).

## 4. Discussion

Strawberries are planted worldwide, and the fruits are highly favored due to their high nutritional content, and they also have high economic value [29]. At present, China has the largest strawberry planting area and the highest yield in the world. However, *F. oxysporum* f. sp. *fragariae* (Fof) can infect strawberries from seedlings to flowering and fruiting plants [36]. Fof invades strawberries through any wound near the roots and proliferates a large number of hyphae. This affects the transportation of water and nutrients, causing old leaves to wither, turn grayish green, and gradually dry [37].

In a previous study, we found that Fof is more pathogenic than Fo. Therefore, in order to explore the reasons for its strong pathogenicity, we located the *SIX* gene and successfully obtained the deletion mutant of *SIX10* [29]. Through experiments, we demonstrated that *∆SIX10* can reduce the pathogenicity of Fof. In this study, we conducted phylogenetic identification of the *SIX9* gene and successfully obtained the deletion mutant *∆Fof16561* of *SIX9*. The analysis indicated the growth rate of *∆Fof16561* is significantly lower than that of the wild-type strain H6 on PDA, CM, MM, and OA media. However, the magnitude of the reduction was relatively small and therefore had no meaningful impact. And, there was no significant difference in spore production and spore germination rate at different stages before complete spore germination. This study suggested that *SIX9* was not involved in growth and development in Fof which was consistent with previous studies [29,38]. In non-biological stress experiments, the growth rate of *∆Fof16561* was significantly faster than that of wild-type strain H6 under conditions of Congo red (for acid stress), KCl (salt stress), and SDS (cell membrane stress), but the increase was relatively small similarly to the status of growth rate. And no significant difference was observed for glucose and NaCl. Therefore, we speculated that the *SIX9* gene did not have important roles in response to these environmental stresses.

Under biotic and abiotic stress, plants activate their antioxidant systems (SOD: superoxide dismutase, POD: peroxidase, CAT: catalase) to clear reactive oxygen species (ROS) such as superoxide anion (O_2_^−^) and hydrogen peroxide (H_2_O_2_), while accumulating osmoregulatory substances (soluble sugars, soluble proteins, proline) to maintain cellular homeostasis. Malondialdehyde (MDA) serves as a lipid peroxidation marker to reflect the degree of oxidative damage. The high activity of SOD, POD and CAT can reduce the invasion of pathogens [39,40,41]. In our research, the deletion mutant *∆Fof16561* can increase SOD and CAT activity in the plant and reduce the pathogenicity of Fof. MDA is a decomposition product of polyunsaturated fatty acids; the rise in MDA suggests that the plants are under oxidative stress, which may result in membrane injury and tissue necrosis [41,42]. In our research, compared to strawberry plants attacked by the wild-type strain, the content of MDA in strawberry plants attacked by *∆Fof16561* was lower after infection for 7 d, which indicated the *SIX9* deletion mutant prevents harm by alleviating membrane lipid peroxidation caused by ROS accumulation. As previous reports reported, excess ROS (i.e., H_2_O_2_ and O_2_^−^) cause lipid peroxidation, followed by destruction of membrane integrity, then result in cell senescence [42,43]. The increase in soluble sugar, soluble protein, and proline content involved in cell osmotic regulation can inhibit the proliferation of pathogens [44,45,46]. The 14 *SIX* genes of Fol have been detected in other formae speciales; however, all 14 *SIXs* have never been simultaneously detected in another single forma specialis [28]. Previous studies showed that SIX genes had important and different functions during pathogenesis and interaction between *F. oxysporum* and host plants, and several *SIX* genes were only expressed in the host plant [21,22,23,24,25]. Research suggested that *SIX* had important roles in different aspects including variability, virulence and host specificity [24,25,26,27,28]. In this study, we speculated that *ΔFof16561* could not trigger as strong a defense response in the plants as the wild-type pathogen, and it was not able to suppress the plant responses as well as the wild type. Moreover, this study indicated the effects of deletion of SIX9 in Fof on pathogenicity in relation to the strawberry varieties and even the tissues (root rot, crown rot, and plant wilt). Multiomics-based approaches are needed to further elucidate this topic [47,48]. And, we speculated that different pathogenic races existed in *Fof* although systematic evaluation was absent until present. In general, the present study provides new insights into the mechanism of action of a *SIX* gene, *SIX9*, in the pathogenicity of *F. oxysporum* on strawberry.

## Figures and Tables

**Table 1:** Primers used in this study.

Primers	Direction	Length	Sequence (5′ → 3′)
*Fof16561*-UP-F	Forward	966	GAGTCCAGTAACGTGGTGCAG
*Fof16561*-UP-R	Reverse		AAAATAGGCATTGATGTGTTGACCTCCGAAACGTTTGTTGTGCTCTTG
*Fof16561*-DOWN-F	Forward	983	CTCGTCCGAGGGCAAAGGAATAGAGTAGCCGGCAGGTGTTCCAGGTC
*Fof16561*-DOWN-R	Reverse		GCCGACGTTG GCCTGGAAG
*Fof16561*-ID-F	Forward	1549	CCAGGTTCCCATAACACGCG
*Fof16561*-ID-R	Reverse		CAGCGGCCCACTCAGCCTG
*Fof16561*-Nest-F	Forward	3086	CCTGATATAAGAGATGAAAC
*Fof16561*-Nest-R	Reverse		GAACTATTCG GCGCTTTCGC
HPH-F	Forward	1349	GGAGGTCAACACATCAATGCCTATT
HPH-R	Reverse		CTACTCTATTCCTTTGCCCT

## Data Availability

Not applicable.
